# The importance of visual features in generic vs. specialized object recognition: a computational study

**DOI:** 10.3389/fncom.2014.00078

**Published:** 2014-08-22

**Authors:** Masoud Ghodrati, Karim Rajaei, Reza Ebrahimpour

**Affiliations:** ^1^Brain and Intelligent Systems Research Laboratory (BISLab), Department of Electrical and Computer Engineering, Shahid Rajaee Teacher Training UniversityTehran, Iran; ^2^School of Cognitive Sciences, Institute for Research in Fundamental Sciences (IPM)Tehran, Iran; ^3^Department of Physiology, Monash UniversityMelbourne, VIC, Australia

**Keywords:** visual features, object recognition, fusiform face area, inferior temporal cortex, face identification

## Abstract

It is debated whether the representation of objects in inferior temporal (IT) cortex is distributed over activities of many neurons or there are restricted islands of neurons responsive to a specific set of objects. There are lines of evidence demonstrating that fusiform face area (FFA-in human) processes information related to specialized object recognition (here we say within category object recognition such as face identification). Physiological studies have also discovered several patches in monkey ventral temporal lobe that are responsible for facial processing. Neuronal recording from these patches shows that neurons are highly selective for face images whereas for other objects we do not see such selectivity in IT. However, it is also well-supported that objects are encoded through distributed patterns of neural activities that are distinctive for each object category. It seems that visual cortex utilize different mechanisms for between category object recognition (e.g., face vs. non-face objects) vs. within category object recognition (e.g., two different faces). In this study, we address this question with computational simulations. We use two biologically inspired object recognition models and define two experiments which address these issues. The models have a hierarchical structure of several processing layers that simply simulate visual processing from V1 to aIT. We show, through computational modeling, that the difference between these two mechanisms of recognition can underlie the visual feature and extraction mechanism. It is argued that in order to perform generic and specialized object recognition, visual cortex must separate the mechanisms involved in within category from between categories object recognition. High recognition performance in within category object recognition can be guaranteed when class-specific features with intermediate size and complexity are extracted. However, generic object recognition requires a distributed universal dictionary of visual features in which the size of features does not have significant difference.

## Introduction

Object recognition is rapidly and robustly performed by human and primate visual system. However, this task is still a real computational challenge for most computer vision systems despite recent amazing progresses (e.g., Serre et al., 2007; Coates et al., 2012; Krizhevsky et al., 2012). We can effortlessly and swiftly recognize virtually unlimited numbers of objects categories even in cluttered backgrounds with changes in illumination, viewpoint, position, and scale. Furthermore, we can simply and accurately recognize objects within a specific category that objects have very similar features (e.g., two similar faces) even in rotated views.

Decades of studies on this remarkable system have revealed that object recognition is performed by the ventral visual pathway (Logothetis and Sheinberg, 1996). Object images, which are first projected on the retina, are spatially sampled based on a cortical magnification factor (Tootell et al., 1982; Van Essen et al., 1984). The sampling resolution is high for objects close to fovea and low in the periphery. Cortical magnification plays an important role in object recognition since the high resolution foveal representation facilitates object recognition and neural receptive field sizes are available both as a function of cortical hierarchy, and as a function of visual eccentricity (Fazl et al., 2009; Grossberg et al., 2011). Visual signals, after retinal processing, are conveyed to lateral geniculate nucleus (LGN), primary visual cortex V1 (Hubel and Wiesel, 1962, 1968), and subsequently to extrastriate visual areas, V2 and V4, and then to the inferotemporal cortex (IT), the projections of visual information finally reach to the prefrontal cortex (PFC) (Perrett and Oram, 1993; Kobatake and Tanaka, 1994). As we trace the pathway from the very first layer such as V1 to the higher processing levels including IT and PFC, the complexity of the preferred stimuli of neurons increase, from simple edges and bars to curves, basic shapes, and finally objects. Also, the size of neurons receptive fields correspondingly increase along this hierarchy (Perrett and Oram, 1993; Kobatake and Tanaka, 1994).

Neurons found in monkey IT cortex have shown that are robust to changes in scale and position of their preferred stimulus (e.g., Logothetis and Sheinberg, 1996; Tanaka, 1996; Brincat and Connor, 2004; Hung et al., 2005). They are also tuned to views of complex objects such as faces (Bruce et al., 1981; Wallis and Rolls, 1997). These neurons can respond distinctively to similar objects within the same category (e.g., two different faces) and remain invariant to changes in scale and position of their preferred stimuli.

A fundamental question in biological object vision is whether the brain utilizes different mechanisms for between categories (i.e., different objects) vs. within category (i.e., faces) recognition or all objects are represented over ventral temporal cortex via distributed patterns of activities distinctive for each object category. The idea that objects are represented over entire ventral temporal cortex via distinctive, distributed patterns of activities is well-supported with functional magnetic resonance imaging (fMRI) (Ishai et al., 1999; Haxby et al., 2001; Spiridon and Kanwisher, 2002; O'Toole et al., 2005; Schwarzlose et al., 2008), optical imaging (Wang et al., 1996; Tsunoda et al., 2001; Yamane et al., 2006), and cell recording studies (Tanaka et al., 1991; Fujita et al., 1992; Kiani et al., 2007; Sato et al., 2013). For example, patterns of responses to cat images have shown to be distinct and highly correlated even in different recording sessions (Haxby et al., 2001). It has also been illustrated that BOLD responses elicited by a set of images from a specific category are significantly correlated with responses to a different set of images from the same category (Spiridon and Kanwisher, 2002). This indicates that distributed patterns of activities have clear information about object categories and are not simply the results of a particular set of images (Spiridon and Kanwisher, 2002; Schwarzlose et al., 2008). Although these results have demonstrated distributed and overlapping patterns of activities in response to different object categories (including faces), other evidence shows that there are restricted areas in visual ventral pathway highly responsive to particular set of objects such as faces (Perrett et al., 1992; Kanwisher et al., 1997; Tsao et al., 2003, 2006; Moeller et al., 2008; Freiwald and Tsao, 2010), places/scenes (Aguirre et al., 1998; Epstein and Kanwisher, 1998; Maguire et al., 1998; Hasson et al., 2003; Kornblith et al., 2013), and bodies (Downing et al., 2001; Pinsk et al., 2005; Van Koningsbruggen et al., 2013). Moreover, recent cell recording evidence from macaque inferotemporal cortex has revealed several islands of entirely face selective neurons (Tsao et al., 2006; Freiwald et al., 2009; Freiwald and Tsao, 2010).

Cell recording evidence indicates that neurons in middle face patch in monkey face selective brain regions respond to face features and combination of these features (Freiwald et al., 2009; Freiwald and Tsao, 2010) which cannot be captured with other IT cells. Features detected by middle face patch are then processed by other anterior face patches (Tsao et al., 2003, 2006) that are responsive to face identities (Moeller et al., 2008; Freiwald and Tsao, 2010). This generally demonstrates that the visual cortex separates generic object recognition (between categories) from within category object recognition (i.e., face recognition). It thus seems that brain employs two different feature extraction mechanisms in the visual cortex for doing these tasks. Computational studies also show that identification tasks (particularly face identification that is holistically Bukach et al., 2010; Piepers and Robbins, 2012; processed-Richler et al., 2011, 2012) need a separate feature set and extraction mechanism to identify, for example, a face (Leibo et al., 2011).

On the other hand, the distributed patterns of activities to a given category have demonstrated discriminative information about the category even when those voxels with maximum responses to the identical category were excluded from the data (Haxby et al., 2001). For example, elicited responses by face images in other visually activated voxels, excluding face patches, showed relatively high discrimination performance for face images (Haxby et al., 2001; Spiridon and Kanwisher, 2002; Tsao et al., 2003). This indicates that visually evoked voxels, outside of face-selective patches, can discriminate between face and non-face objects categories and face patches are required to discriminate fine differences in within category object recognition (i.e., face identification-see: Freiwald et al., 2009; Freiwald and Tsao, 2010).

Since within category object recognition requires to individuate highly similar objects and features, it can be suggested that cortex uses an expert module (Gauthier et al., 1999, 2000a; Harel et al., 2010; Bilalić et al., 2011; McGugin et al., 2012) capable of extracting specialized visual features and employing these features to discriminate between similar objects within a category. Therefore, the type of extracted features plays an important role here.

Here we computationally analyze the role of visual features and extraction mechanism in between and within category object recognition. We study the results of two biologically plausible object recognition models (one is our previous work introduced by Rajaei et al., 2012 and the other well-known HMAX model-Serre et al., 2007; Riesenhuber and Poggio, 1999) in within and between categories object recognition tasks. Briefly, the proposed model extracts a set of visual features using an unsupervised learning method, inspired by biology, while the HMAX model extracts a random set of features in the learning stage (Serre et al., 2007). The unsupervised feature learning makes the model capable of extracting features without any constraint [e.g., considering an objective function to lead higher classification performance (Ghodrati et al., 2012) or information maximization (Ullman et al., 2002)]. This thus allows us to study the types of features extracted with the model in different recognition tasks. Overall, the results show that despite models differences, the visual features are important factors in recognition tasks.

To explore the role of visual features in between and within category object recognition, we selected face images from different identities for within category recognition task. Faces are intensively studied in computational (e.g., Turk and Pentland, 1991; Brunelli and Poggio, 1993; Belhumeur et al., 1997; Leibo et al., 2011; Tan and Poggio, 2013) and experimental studies (Diamond and Carey, 1986; Gauthier and Logothetis, 2000; Gauthier et al., 2000b; Maurer et al., 2002; Tsao et al., 2006; Pitcher et al., 2007; Robbins and McKone, 2007) and are agreeable model category for within category recognition. We also used Caltech-256 to study between category object recognition (Griffin et al., 2007).

The results show that face identification, as a within category object recognition task, not only requires class-specific features, extracted from individual faces, to distinguish between very similar objects with fine differences in features within a class (expertise), but we also need to increase the size of prototype (a patch of an image that cover partial object view) up to intermediate sizes to achieve higher recognition performance (that can be referred to holistic processing). However, in between object recognition there is no considerable difference in recognition performance either when class-specific features are extracted or the prototype size increases. This supports the idea that in between category object recognition, a dictionary of visual features that contains many features of real-world objects can handle the task (distributed model).

## Materials and methods

### Biologically inspired models

#### Stable model

The model, which was previously introduced by our group (Rajaei et al., 2012), is a biologically motivated object recognition model which employs a learning method inspired by the Adaptive Resonance Theory (ART-Grossberg, 2013) in feature extraction stage. Generally, the proposed model has a hierarchical structure. The ART mechanism was used in the model for extracting more informative visual features of intermediate complexity, and this consequently provides a more realistic biologically inspired model. The proposed model, by utilizing a hierarchical structure, intends to emulate processing performed in the ventral visual pathway.

**Figure 1:**
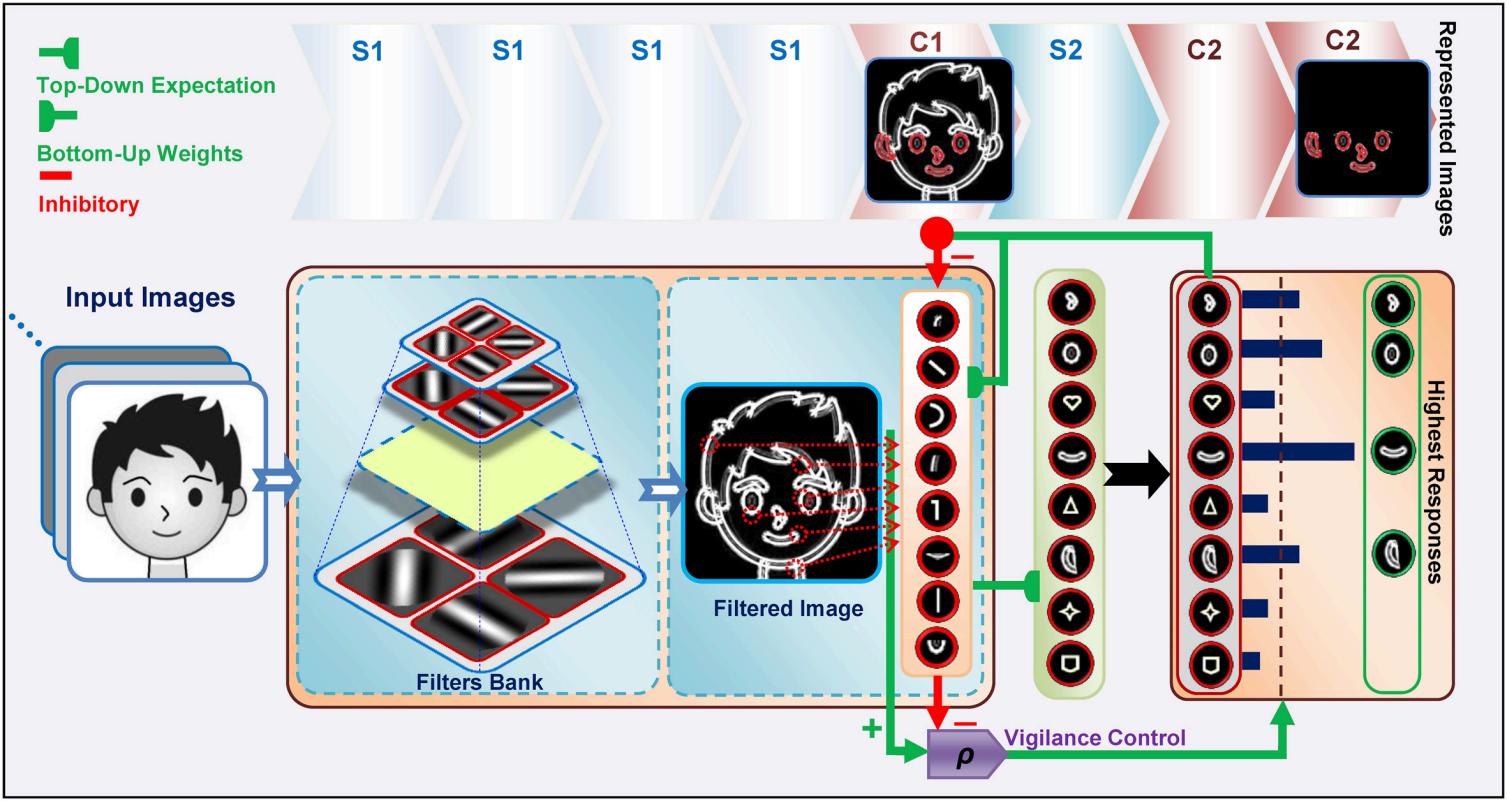
**The structure of the Stable model**. Gray scale images are applied to the model and the outputs of S1 and then C1 are attained. Then, the S2 responses are computed using existing prototypes. To compute the C2 responses, the S2 units with the maximum response for each prototype are selected. The highest active C2 units are then selected as prototypes to represent the image (see green box at right, specified by “Highest Responses”). This selection is achieved by top-down expectations, which match the input image to the prototypes. A lateral subsystem (vigilance control), which uses a vigilance parameter (ρ), determines the matching degree between the prototypes and various parts of the input image.

**Figure 2:**
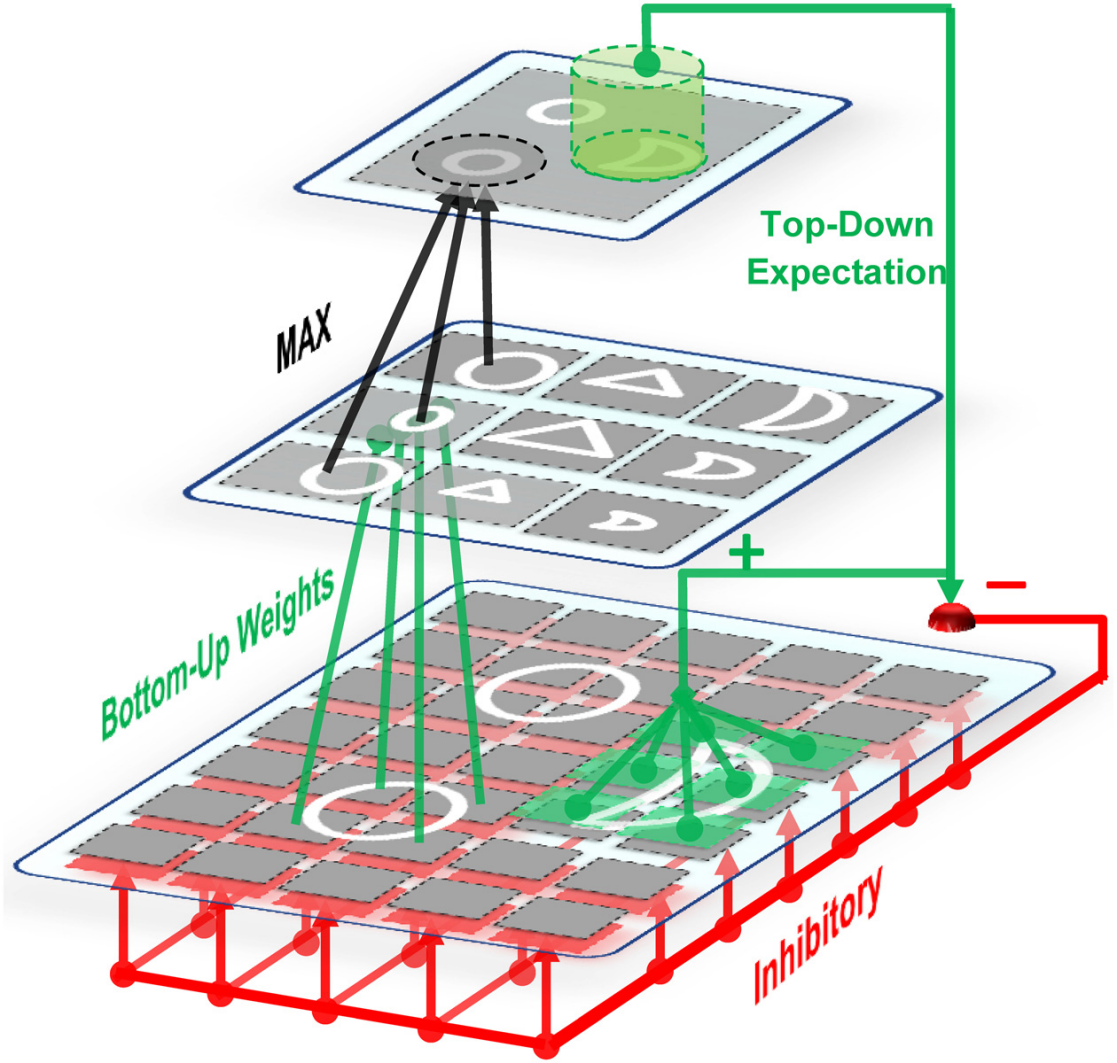
**On-center off-surround network**. Some units in the upper layer are activated by bottom-up weighted connections. Excitation signals are sent via these units to the relevant units through direct top-down weights and inhibition signals are sent to all units. This amplifies the activities of cells within the matched (on-center) portion while suppressing the activities of irrelevant cells in the non-matched (surround) portion. Therefore, this network is named the on-center, off-surround network. The units in the first layer receive both excitation and inhibition in which additional excitations may overcome the inhibitions. In contrast, when the cells receive only top-down inhibition (off-surround), then one inhibition may counteract one excitation from the input.

Images are processed with four consecutive layers of simple and complex units, Figure 1. The first layer in the model contains units that extract bars and edges from input images (S1 units). These units take the form of Gabor function (Gabor, 1946) and convolve the input images with filter windows to detect bars and edges. The responses of complex units in C1, which is the next layer, are acquired by max pooling over a group of simple S1 units which have the same preferred orientation but at slightly different positions and sizes (Serre et al., 2007). This pooling increases the invariance to the changes in shift and size inside the receptive field of the units. In the next layer, named S2, more complex patterns than bars and edges are represented within the receptive field. The units of this layer receive their inputs from retinotopically organized C1 units in a spatial grid via weighted connections that respond to specific patterns or prototypes, bottom-up weights, Figure 2. The C2 is the last layer of the model that responds to the prototypes of the input image extracted from different locations. There are connections between a C2 unit and several S2 units of the same prototype, but in different sizes and positions. Therefore, the results of this layer are C2 values in a vector of size N, where N is the number of prototypes learned by the model.

C2 responses illustrate the matching degree between the prototypes and the input image. When we have a high C2 response, this indicates that the extracted prototype is sufficiently matched by a portion of the input image and is thus suitable for representing the input image (for more information see: Rajaei et al., 2012). In addition to feed forward connections, there are feedback connections from complex to simple units which simulate feedback from complex cells to simple cells through the on-center, off-surround network in the V1 and V2 areas of the visual cortex, Figure 2. This makes a feedback loop that yields a resonant state for relevant cells (Bullier et al., 2001). The match learning between input and output was simulated based on this feedback loop to learn informative intermediate-level visual features from the input images. This feedback excites portions of inputs that are matched by the prototypes of the active C2 units and inhibits portions of inputs that are not matched by these prototypes (Figure 2—for more information refer to Rajaei et al., 2012). To achieve informative prototypes for each image, we employed the match learning and reset mechanism of the ART system, Figure 1.

The training phase was performed by presenting all of the training images to the model, and continued by attaining outputs of S1 and then C1. The S2 responses were then computed by utilizing the existing prototypes. Next, to compute the C2 responses, the S2 units with a maximum response for each prototype for all of the positions and scales were selected. Then P number of C2 units with the highest activity was selected for better representation of the input image (this selection and matching procedure between the input image and prototypes were achieved by top-down expectations, Figures 1, 2. Here P was set to 5 which is based on our previous study, Rajaei et al., 2012) and then compared them with a vigilance parameter to characterize the matching degree between the prototypes and the input image. The selected units are schematically shown at the C2 level in Figure 1. If the vigilance control determines that the amount of matching is low, then the current prototype is not appropriate to represent the input image. Consequently new prototypes are extracted from the current input image and added to the prototype pool. Using this learning process, with a single presentation of an image from the training set, proper prototypes that effectively represent the image are extracted.

After extracting informative features in training phase, the features set was used in test phase. For all images in testing sets, each image was passed through the layers of the model and the responses of the C2 units were computed and saved as a vector representing the extracted features for that image. Next, these vectors were subsequently passed to a linear classifier (i.e., linear SVM) for classification.

#### HMAX model

In this study we used feed forward, four-layer version of HMAX model proposed by Serre et al. (2007). Briefly, after convolving input images with a set of Gabor filters in the S1 layer, C1 maps are built up by max pooling over S1 responses. At training phase, a large set of image patches are randomly extracted from C1 maps. These patches are used as the center of Gaussian-like functions in which the distance of the input test image with these patches is computed. This procedure yields S2 maps. The C2 responses are subsequently obtained by taking a global max over all S2 responses to an input image. The C2 feature vectors are then applied to a linear SVM classifier.

### Image database

We used Caltech-256 image database for between category object recognition task (Griffin et al., 2007) and PIE face database (Sim et al., 2002) for within category object recognition task. The CalTech-256 in total contains 30,607 images from 256 different object categories. The minimum number of images in any category is 80 and the maximum is 827 images. In each run and experiment, we randomly selected a subset of object categories and images from the database (see the Results). The PIE face image database consists of 41,368 images taken from 68 different people. Photographs were taken in different lightening conditions, poses, and face expressions. We used images of different identities for face identification task (within category).

### Experiments

The performance of the models was evaluated in object categorization (between category object recognition) and face identification tasks (within category object recognition). In the object categorization tasks, models were trained using 30 images and tested using 50 images from each object category. Performances were calculated for different number of object classes (2, 5, 10, 20, 30, and 40-multiclass) and different patch sizes (4^*^4, 8^*^8, 12^*^12, 16^*^16, 20^*^20, 24^*^24). In each run a number of object classes were randomly selected. For example, we randomly selected 10 object categories out if 256 classes. Subsequently, each category were randomly divided to train and test images (i.e., 30 train and 50 test images for object categorization task). All images were converted to gray-scale and the height of images was resized to 140 pixels while the aspect ratio was preserved. The same selection procedure was considered in face identification tasks.

**Figure 3:**
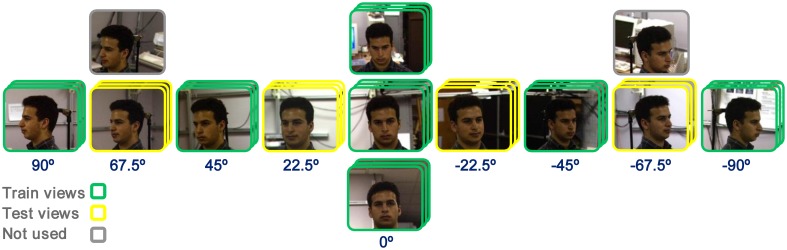
**Image selection for face identification task**. Views of 0, ±45°, and ±90°, with three images in each view (expression), were used as train images (specified with green frame). Views of ±22.5° and ±67.5° were selected as test images (specified with yellow frame) and some other images were not used in experiments (gray-frame images).

In face identification tasks 21 and 12 face images from each identity were used as training and test images, respectively. Similarly, the performances were calculated for different number of identities (10, 20, 30, and 40-multiclass) and different patch sizes (4^*^4, 8^*^8, 12^*^12, 16^*^16, 20^*^20, 24^*^24). In each run, a number of identities were randomly selected. Face images of views 0, ±45°, and ±90° were presented to the models as train images and other views were used in test phase (±22.5° and ±67.5°). To increase the number of images in face identification task, we used some expression in each view. The selection of face images are illustrated in Figure 3. The performances in all experiments and plots are generally the results of 15–30 independent random runs and the mean and standard deviation (SD) are reported. Some results are reported using boxplot. The reported performances are the percentage of correct responses (recognition rate). It is worth noting that the patches, which are extracted in intermediate layers in models, are defined in terms of proportion of a whole face/object they cover, not in terms of degrees of visual angle. For example, in our study, considering the size of images, a patch with the size of 12^*^12 covers a partial view of a face while a patch of size 16^*^16 or 20^*^20 covers a whole view or more. This was controlled prior to main experiments.

As mentioned earlier, the experiments were performed in three different feature learning modes that are described in following sections.

#### Within feature learning

**Figure 4:**
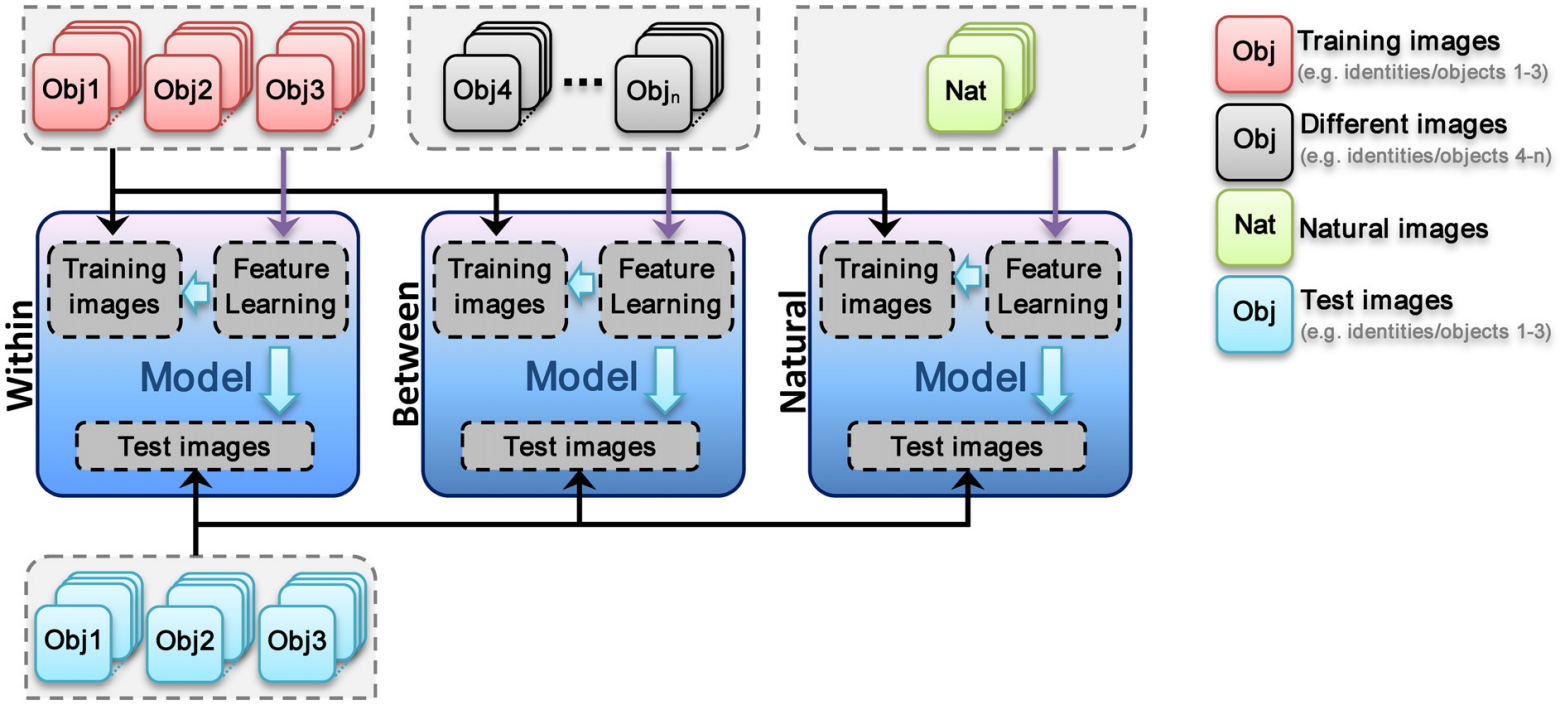
**Different modes of visual feature learning**. In *within feature learning* mode visual features are learned using train images (train and test images are the same identities or object categories but images are completely different in both sets). In the second mode (*between*), a different set of identities or object categories are selected for feature learning stage. In the third mode, a large set of natural images are diversely selected from the web. This set are then used to learn visual features. In each mode, the images for feature learning are only changed.

In this mode, visual feature are learned using training images, which have the same categories/identities as test images but different images. For example, in face identification task, the images of three identities are randomly selected and then divided to test and train sets. Subsequently, models use train images to learn visual features from images with identical identities to test images but with different views from test images, Figure 3. The learned visual features are then used in test phase. The procedure is schematically represented in Figure 4 for all three different types of experiments (*Within, Between*, and *Natural*). This experiment allows us to assess the performance of models when class-specific features are extracted (both for object categorization and face identification).

#### Between feature learning

In this mode, visual features are learned using different identities/categories from training and test images. For example, in face identification task, the images of three identities are randomly selected and then divided to test and train sets. Following that, a different group of face identities are randomly selected and models use these images for visual features learning stage. As mentioned these images are completely different identities or object classes from test and train images. The learned visual features are then used in test phase (see Figure 4). This feature learning strategy helps us to investigate the role of features, extracted from the same categories but different identities, in recognition performances. For example, in face identification task, all patches in learning phase are extracted from face images but different identities from test and train images. This can help us to understand whether visual features, extracted from the same category, can be generalized to identification tasks of identical categories/classes.

#### Natural feature learning

In this mode, a large number of natural images were selected from the web (see Figure S1 for sample images from natural image set). Models use this images set to learn visual features. Authors tried to select natural images as diverse as possible. Images contain both indoor and outdoor scenes in which some images include one or several objects. The learned visual features are then used in test phase (see Figure 4). Using these features, we aim to explore whether a large dictionary of visual features (~6000 patches in different sizes), learned from a diverse set of images; can solve the problem of object categorization and how these features act in identification tasks.

## Results

We have selected the Stable model (Rajaei et al., 2012) and HMAX model (Serre et al., 2007) to examine how different visual features perform in specialized vs. generic object recognition (face identification vs. object categorization). The HMAX model randomly extracts a set of image patches with intermediate complexity from C1 maps. These patches can be analogous to the preferred stimuli of V4 and some IT neurons in primate visual cortex (Tan and Poggio, 2013). Using these randomly selected patches, the HMAX model constructs S2 and C2 feature through a hierarchical structure. Finally, C2 features are classified in a supervised manner. However, the Stable model utilizes an ART-based mechanism to learn visual features in an unsupervised manner. This enables the Stable model to learn more informative features (more details in Materials and Methods). This model selection allowed us to evaluate the performance of randomly selected features (i.e., HMAX model) and an unsupervised feature learning mechanism (i.e., Stable model) in two different tasks of object recognition. We found that both models performed similarly in these tasks, regardless of using random features or unsupervised method. We analyzed the performance of different visual features using two object recognition models in two hotly-debated object recognition problems in the following sections.

### Face identification

**Figure 5:**
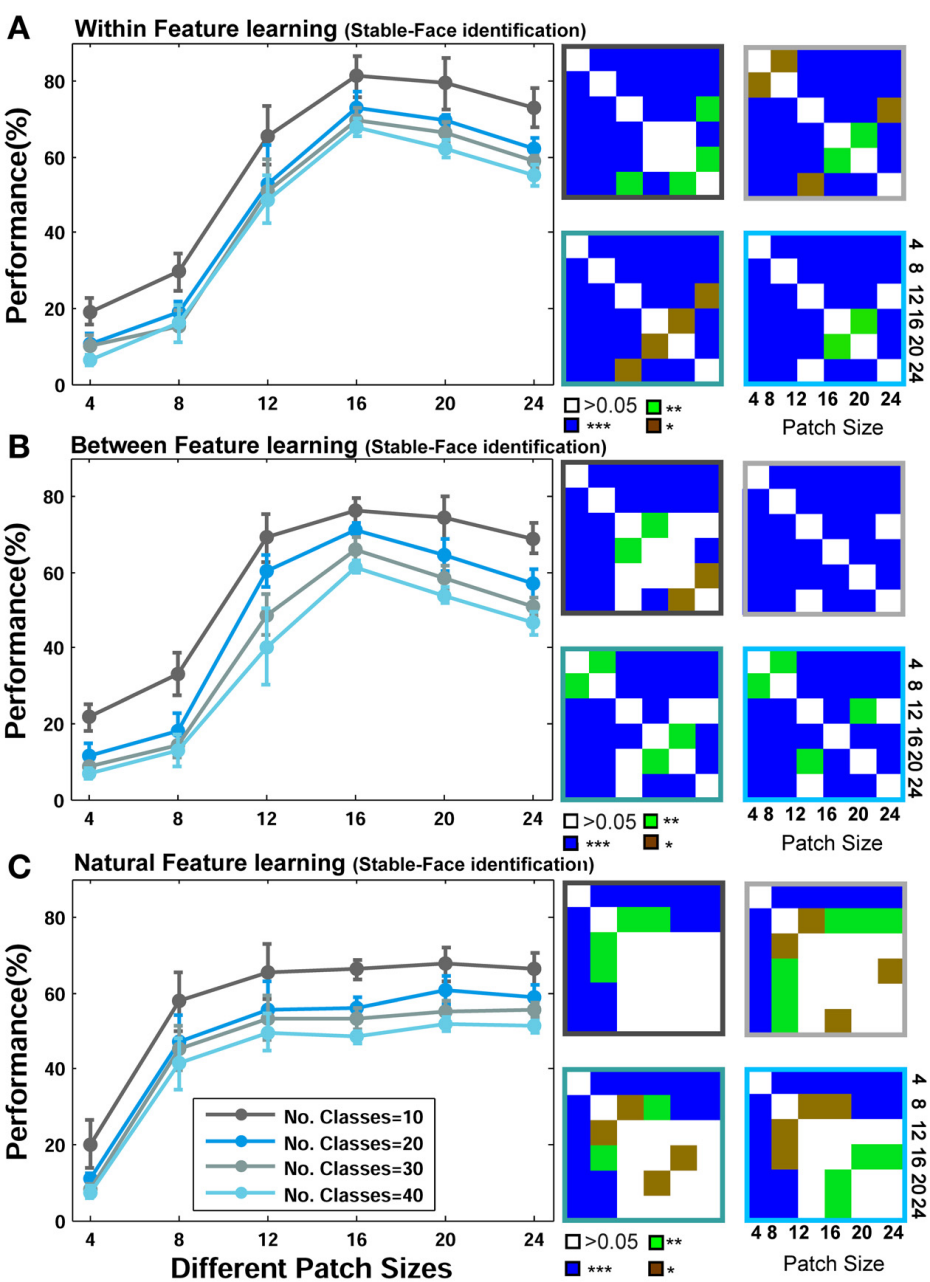
**Performance comparisons between different patch sizes and feature learning strategies for the Stable model in face recognition task. (A)** Comparing the performance of the Stable model when uses face images from identical identities to test images but with different views in feature learning phase. Each curve shows the results of different number of classes/identities that vary from 10 to 40 (specified with different colors). The results are the average of 15 random runs and error bars are standard deviation. Right insets, next to each plot, demonstrate *p*-values for all possible comparisons within each experiment. The color code shows the significance level and the color of the frames corresponds to the related curve (number of classes). Each symbol shows a *p*-value: “^*^” for *p* < 0.05, “^**^” for *p* < 0.01, and “^***^” for *p* < 0.001. **(B)** Performance comparison when model employs different face images for feature learning. **(C)** Performances when features are learned from a large set of natural images.

**Figure 6:**
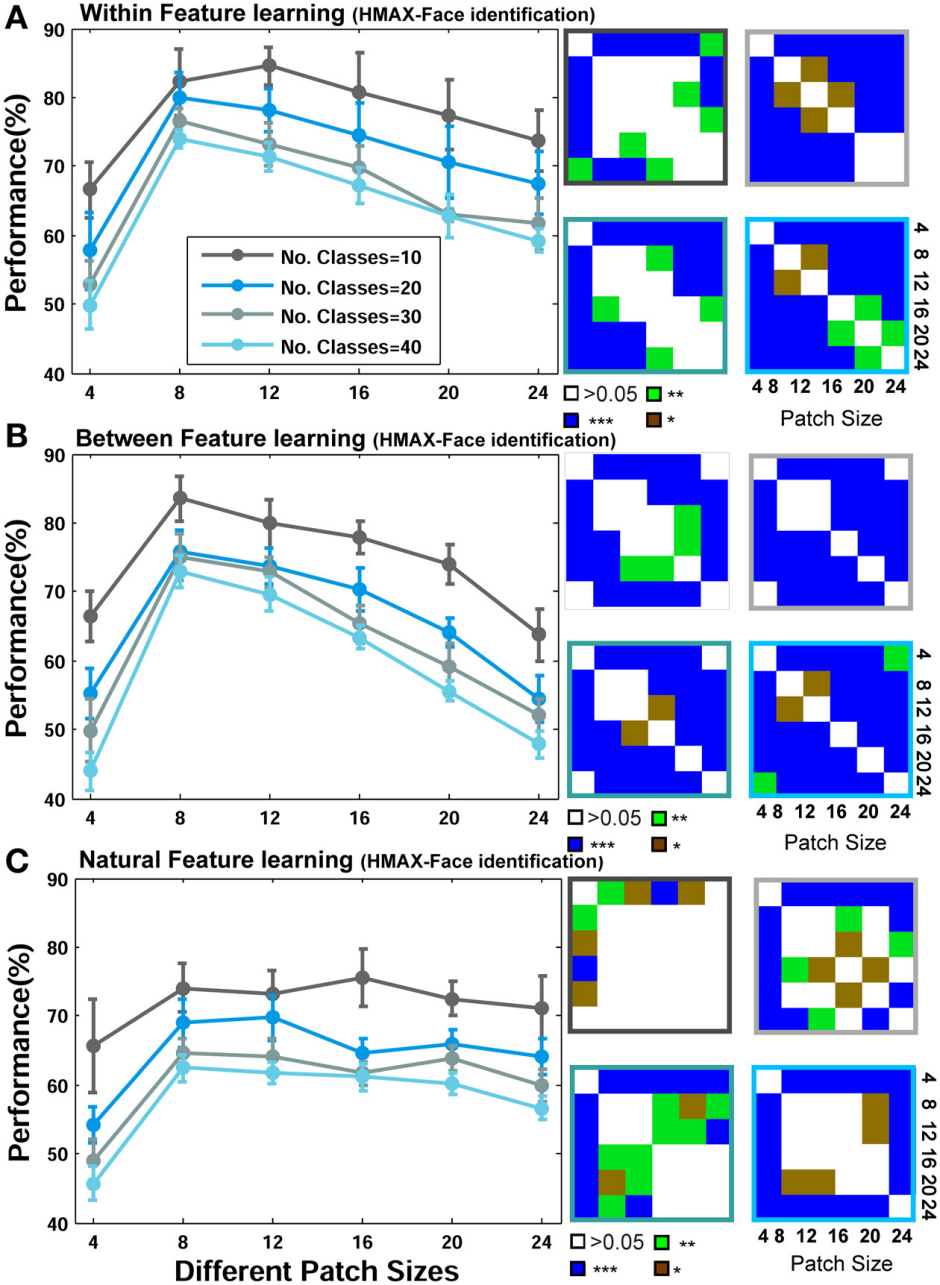
**Performance comparisons between different patch sizes and feature learning strategies for the HMAX model in face recognition task. (A)** Comparing the performance of the HMAX model when uses face images from identical identities to test images but with different views in feature learning phase. Each curve shows the results of different number of classes/identities that vary from 10 to 40 (specified with different colors). The results are the average of 15 random runs and error bars are standard deviation. Right insets, next to each plot, demonstrate *p*-values for all possible comparisons within each experiment. The color code shows the significance level and the color of the frames corresponds to the related curve (number of classes). Each symbol shows a *p*-value: “^*^” for *p* < 0.05, “^**^” for *p* < 0.01, and “^***^” for *p* < 0.001. **(B)** Performance comparison when model employs different face images for feature learning. **(C)** Performances when features are learned from a large set of natural images.

We initially present the results of face identification experiment, as a within category object recognition task, for different feature learning strategies (*Within, Between*, and *Natural* visual features). The performances are the results of 15 random splits and the mean and standard deviation (SD) are reported. We used a linear SVM classifier to obtain the performances. To statistically test the significance of the results, we used Wilcoxon rank sum test and reported *p*-values for different comparisons. The recognition performances for different patch sizes and number of classes/identities are demonstrated in Figure 5 (for the Stable model) and Figure 6 (for the HMAX model).

As represented in Figures 5A, 6A, when models learn visual features from faces of identical identities to test images but with different views (*Within category feature learning*, see Materials and Methods—Figure 4), the performances are generally higher than two other learning strategies: when models learn features from different identities, Figures 5B, 6B (*Between category feature learning*) or from a group of natural images, Figures 5C, 6C (*Natural visual feature learning*). This result is roughly evident for all patch sizes and number of classes. We draw closer comparisons between performances of different training modes in other parts of the paper. The right insets in Figures 5, 6 (also see **Figure 9**) show the *p*-values for different comparisons between performances. For example, we compared the performance of the Stable model, when tested using 10 classes/identities, in different patch sizes. We drew all possible comparisons in this case (e.g., performance of patch size 4 with 8, 4 with 12, etc.) to show whether the increases and decreases in performances are significant. This yielded a *p*-value matrix of size 6^*^6 (6 patch sizes). The color code shows the significant level.

The second interesting result that can be seen in Figures 5, 6 is higher performance of patches with intermediate sizes in recognizing face images of different identities. Almost in all modes of feature learning, the performance of models is poor in recognizing faces when models only use features of size 4^*^4. This occurs due to the small area that a patch of this size covers in a face image. A patch of size 4^*^4 only contains a very small part of an image and does not provide sufficient information about a face or components of a face image (e.g., nose, eye, or mouth). It is very difficult for models to distinguish between individual faces or within object categories using only small patch sizes (i.e., 4^*^4) because they lack the selectivity required to encode fine differences. However, as the size of patches increases to more intermediate sizes (12, 16, 20 for the Stable model and 8, 12, 16, 20 for the HMAX model), the performance of the models elevates significantly (see *p*-values for all possible comparisons in the right insets). This is evident either when features are learned from identical face images to test images, Figures 5A, 6A (*Within category feature learning*- more evident with the highest performance among all modes) or when models use different face images for learning visual features Figures 5B, 6B (*Between category feature learning*). The results, however, are different when models employ natural images for learning visual features Figures 5C, 6C. There is approximately no significant difference between the performances of intermediate patch sizes in face identification task in *natural feature learning* strategy. This indicates that for within category object recognition we need to extract class-specific visual features with appropriate sizes to cover a whole or partial view of an object (here faces) to achieve high performance. This is an essential factor that makes the models, and perhaps primate visual cortex, capable of recognizing fine differences between highly similar objects (i.e., faces) and this task cannot be accomplished with a dictionary of visual features learned from a large set of natural images. Moreover, an analogy can be drawn between class-specific visual features and the concept of expertise in visual cortex (discussed in Introduction and Discussion).

**Figure 7:**
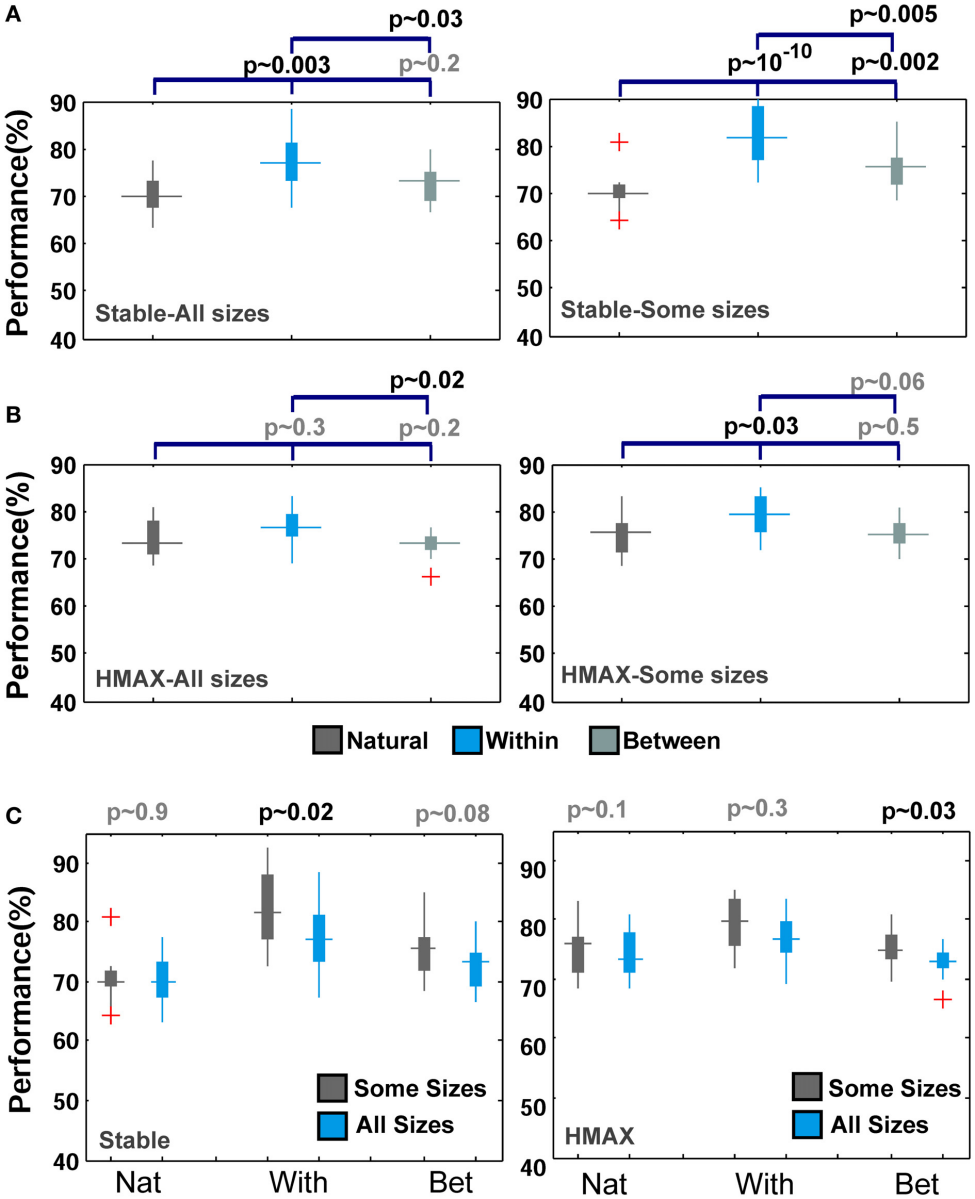
**Comparing the performance of the Stable model as well as HMAX in different feature learning strategies in face recognition task**. (**A**-left) The performance of the Stable model when all patches contribute to overall performance in face identification task for 10-class identification task. The dark gray boxplot shows the performance of the model when feature are learned from natural images. Pale gray boxplot represents the performance once model uses a different set of face images for learning visual features and the cyan boxplot illustrates the performance of *within category feature learning* strategy. *P*-values are shown on the top of each plot for all possible comparisons. (**A**-right) The performance of the Stable model when intermediate patch sizes contribute to overall performance in face identification task. The red crosses in plots are outliers. (**B**-left) The performance of the HMAX model when all patches contribute to overall performance in face identification task (10 classed). (**B**-right) The performance of the HMAX model when intermediate patch sizes contribute to overall performance in face identification task. (**C**-left) Comparing the performance of the Stable model in face identification task for all patch sizes (cyan boxplots) and intermediate patch sizes (gray boxplots) for different learning strategies (Nat, With, Bet). (**C**-right) Comparing the performance of the HMAX model in face identification task for all patch sizes (cyan boxplots) and intermediate patch sizes (gray boxplots) for different learning strategies.

Figures 5, 6 mostly illustrate the performance comparison between various patch sizes (from small to intermediate and upper-intermediate) in three feature learning modes but they do not draw a clear comparison between the performances in different feature learning strategies. We asked whether the performance significantly differs between these three training strategies when we average the contribution of all patches into the overall performances, regardless of particular sizes. These results are shown in Figure 7A-left (for the Stable model) and Figure 7B-left (for the HMAX model). We only compared the results for the case of 10-class face identification. As can be seen, even when all patch sizes contribute to the overall performance, the performance of both models is higher in the case of *within category feature learning* than two other learning modes (*Between* and *Natural feature learning*. *p*-values are depicted on the top of each plot). Here we reported the results using boxplot method to provide more clear comparisons between learning strategies. These results confirm the idea that specialized object recognition can properly be performed with specialized features (class-specific) rather than a large set of different features.

Closer inspection reveals that higher recognition performance can be achieved when we omit small patch sizes and average the overall performances of intermediate sizes; see Figures 7A,B (right column). This again indicates that for face identification, we need to extract class-specific features with intermediate sizes. Figure 7C represents better comparison once all patch sizes contribute to overall performance and when we only consider intermediate sizes. As can be seen, the performance significantly differs when only intermediate sizes are considered, Figure 7C.

**Figure 8:**
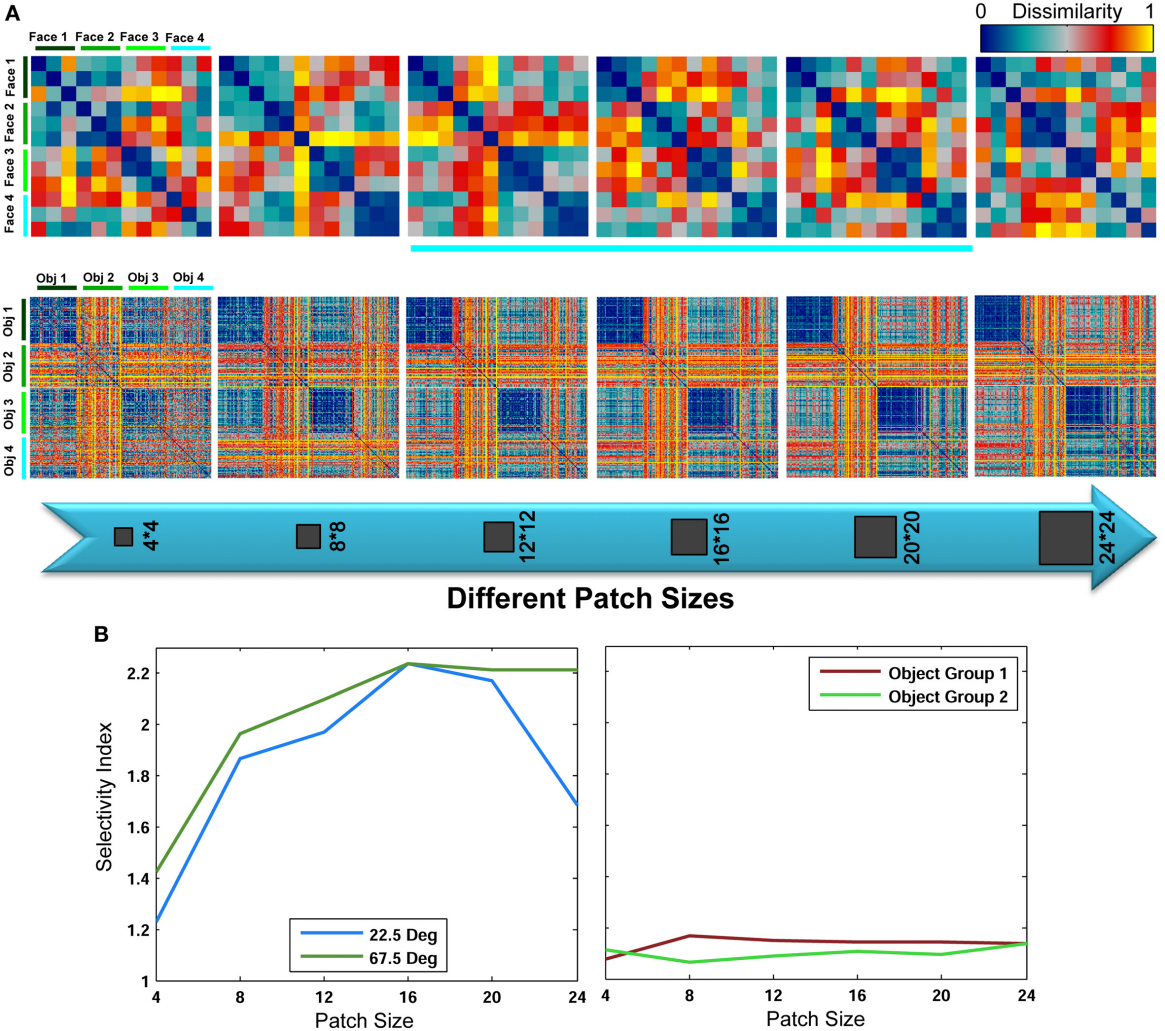
**Representational dissimilarity matrices for different patch sizes, computed for the Stable model**. (**A**-top row) Dissimilarity matrices for face identification task averaged over 15 random runs for different patch sizes. Each matrix shows the dissimilarity between feature vectors of four different identities of a test view of 67.5°. The matrices are the size of 12^*^12 because there were three face images for each angle (here 67.5°), see Figure 3. Each 3^*^3 square along the diagonal line represents the dissimilarity between features of one identity. These RDMs were only computed for *within category feature learning* strategy. (**A**-bottom row) Dissimilarity matrices for object recognition task averaged over 15 random runs for different patch sizes. Each matrix shows the dissimilarity between feature vectors of four different objects. The matrices are the size of 200^*^200 because there were 50 object images for each category. Each 50^*^50 square along the diagonal line represents the dissimilarity between features of one object category. **(B)**. Selectivity index calculated based on RDMs for different patch sizes for face (view 22.5° and 67.5°) and objects (two groups of 4 objects).

These results reveal that for face identification, class-specific visual features with intermediate sizes yield significantly higher classification performances compared to a large dictionary of visual features. An important question is why patches with intermediate sizes result in higher performances and small patch sizes fail to achieve this level of performance? As briefly described earlier, a key reason is that intermediate sizes cover larger areas of a face in an image; therefore, they are more informative about a face and can recognize fine differences more accurately. But what occurs in features space, when intermediate sizes are only considered, that makes this task easier for classifier (here linear SVM). One idea is that using intermediate sizes in face identification task causes features of different identities cluster close together in feature space; due mainly to the amount of information about a face identity that a patch with intermediate size contains, and this increases the discriminability and makes the feature space less complex for classifier. To address this, we constructed representational dissimilarity matrices (RDMs) based on dissimilarity between feature vectors of all images in all categories/face identities (measured as 1-correlation, see: Kriegeskorte et al., 2008; Kriegeskorte and Gabriel, 2012). Using four different identities and representational dissimilarity matrices for each patch size, we tried to visualize the feature space, see Figure 8 (RDMs were only computed for the Stable model and in *within feature learning* mode using RSA toolbox-Nili et al., 2014). As Figure 8A (first row) shows, as the size of patches increases from small to intermediate sizes, the face images of each identity are clustered together more clearly and the similarity (or dissimilarity for 1-correlation) of features within each identity increases within their cluster (the blue squares along the diagonal line which is more clear for intermediate sizes than other sizes). This indicates that intermediate sizes contain important information about a whole face or a partial view of a face that enables the models to discriminate between fine differences in face identification task. To generate a quantitative measure for the differences in the RDMs of various patch sizes, we used selectivity index that is obtained by dividing the average of pixel values within categories/identities in the RDMs (squares along diagonal line) to the average of other remaining pixel values in RDMs (the diagonal pixels were disregarded from calculation). To calculate selectivity index, we used correlation values instead of 1-correlation. A perfect selectivity is achieved when all pixel values in diagonal squares have value of 1 and other pixels 0 (1/0 = infinite). The results are shown in Figure 8B for object and face recognition. These results also confirm that the size of patches do not have significant effects in generic object recognition while face identification needs intermediate patch sizes.

Taken the results into account, to perform specialized object recognition, models and visual cortex require distinguishing between highly similar features (e.g., faces of two identities) and this cannot be accomplished without considering highly selective unites in models (neurons in visual cortex) responsive to different face identities. In computational models, one approach to increase selectivity of units is enlarging the tuning sizes of units (here patch sizes—Tan and Poggio, 2013). This approach is similar to the concept of holistic processing for faces in visual system.

### Object recognition

To analyze the performance of the models in generic object recognition, we randomly selected a number of object categories from CalTech-256 image database (Griffin et al., 2007) in each experiment and ran the models using images of selected categories (Figure S2 shows several sample images for both databases). Due to the high diversity of object categories and images in each category, each experiment was performed for 30 independent random runs for different number of classes (2, 5, 10, 20, 30, and 40) and the mean and standard deviation are reported. This provides us with more reliable performances (mean and STD) in object recognition task. In several plots we report the results using boxplot method.

Similar to face identification experiments, we first report the performances of different patch sizes in two feature learning modes. In object recognition experiments, models learned visual features in two strategies: *natural feature learning* in which models use a large set of natural images containing a wide variety of objects in learning phase and *within category feature learning* in which visual features are learned from the same categories as test images but with different images (Refer to Materials and Methods-Figure 4). We did not use *between category feature learning* because this is very similar to *natural feature learning* in the concept of generic object recognition.

**Figure 9:**
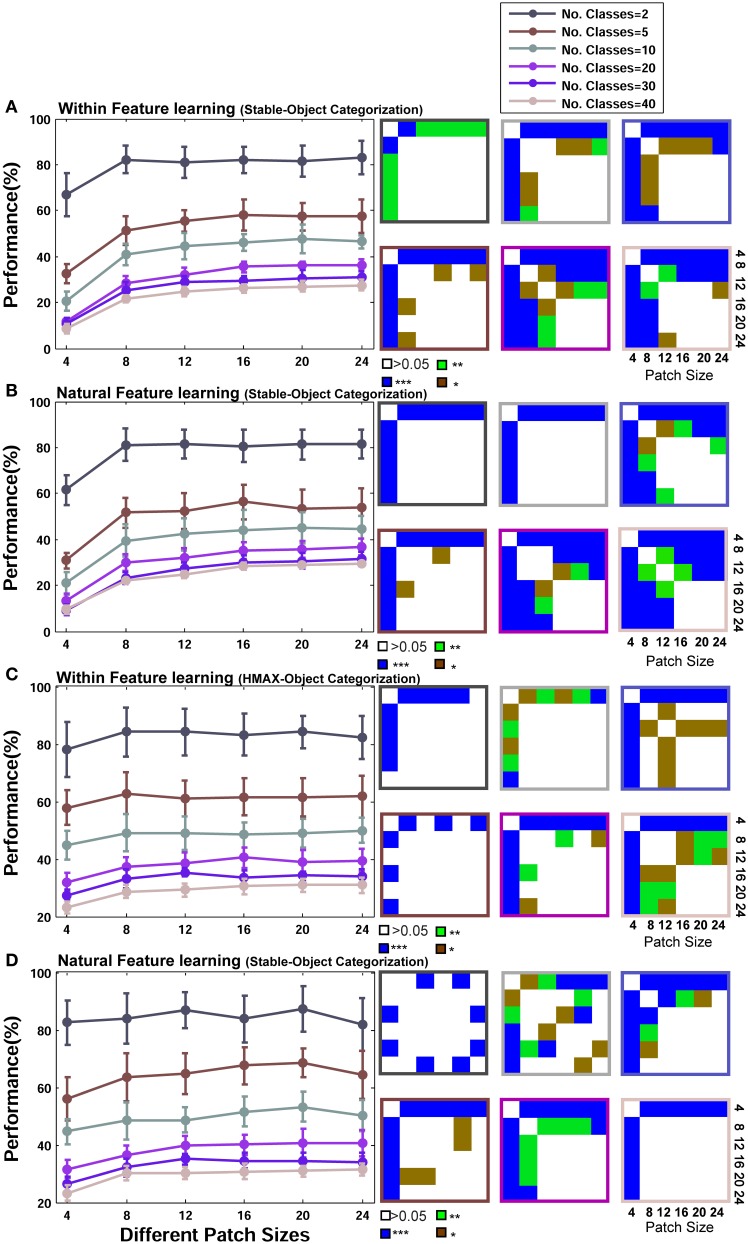
**Performance comparisons between different patch sizes and feature learning strategies in object recognition task**. **(A)** Performance comparison between different patch sizes for the Stable model when uses natural images in feature learning phase. Each curve shows the result of different number of classes that vary from 2 to 40 (specified with different colors). The results are the average of 30 random runs and error bars are standard deviation. Right insets, next to each plot, demonstrate *p*-values for all possible comparisons within each experiment. The color code shows the significance level and the color of frames corresponds to related curves (number of classes). Each symbol shows a *p*-value: “^*^” for *p* < 0.05, “^**^” for *p* < 0.01, and “^***^” for *p* < 0.001. **(B)** Performance comparison when Stable model selects *within category feature learning* strategy. **(C)** Performance of the HMAX model for different patch sizes when features are learned from a large set of natural images. **(D)** Performance comparison when the HMAX model selects within category feature learning strategy.

Figure 9 represents the results of object recognition experiments for different sizes of patches and number of classes for both the Stable model and the HMAX model. Overall, the difference in performances of different patch sizes is not considerable, although significant, compared to the same condition in face identification (*p*-values for all possible comparisons are shown in right insets). Figure 9A illustrates the results of the Stable model once features are learned from natural images and Figure 9B shows similar results when the model uses *within category feature learning*. This shows that increasing the sizes of patches does not considerably increase the recognition performance in object recognition since this task does not require highly selective unites. It can also be seen that the performance of the model in two different learning strategies is not significantly different in all patch sizes and number of classes (more detailed comparison is presented in following parts of the papers). This result indicates that for the task of generic object recognition, a visual dictionary of features can perform well and it makes no significant difference if models extract class-specific features. The only significant difference is for patches of size 4^*^4 (this is more significant for the Stable model). This happens for two reasons: first, this small size is not informative enough for classifying different categories. Second, the Stable model uses an unsupervised feature learning mechanisms and tries to extract dissimilar/decorrelated patches (see Materials and Methods); this thus forces the model to learn a few numbers of patches with this size due to the low diversity of 4^*^4 patches. This thus yields poor classification performance.

Figures 9C,D similarly show the results of the HMAX model. These results are very similar to the results of Stable model.

**Figure 10:**
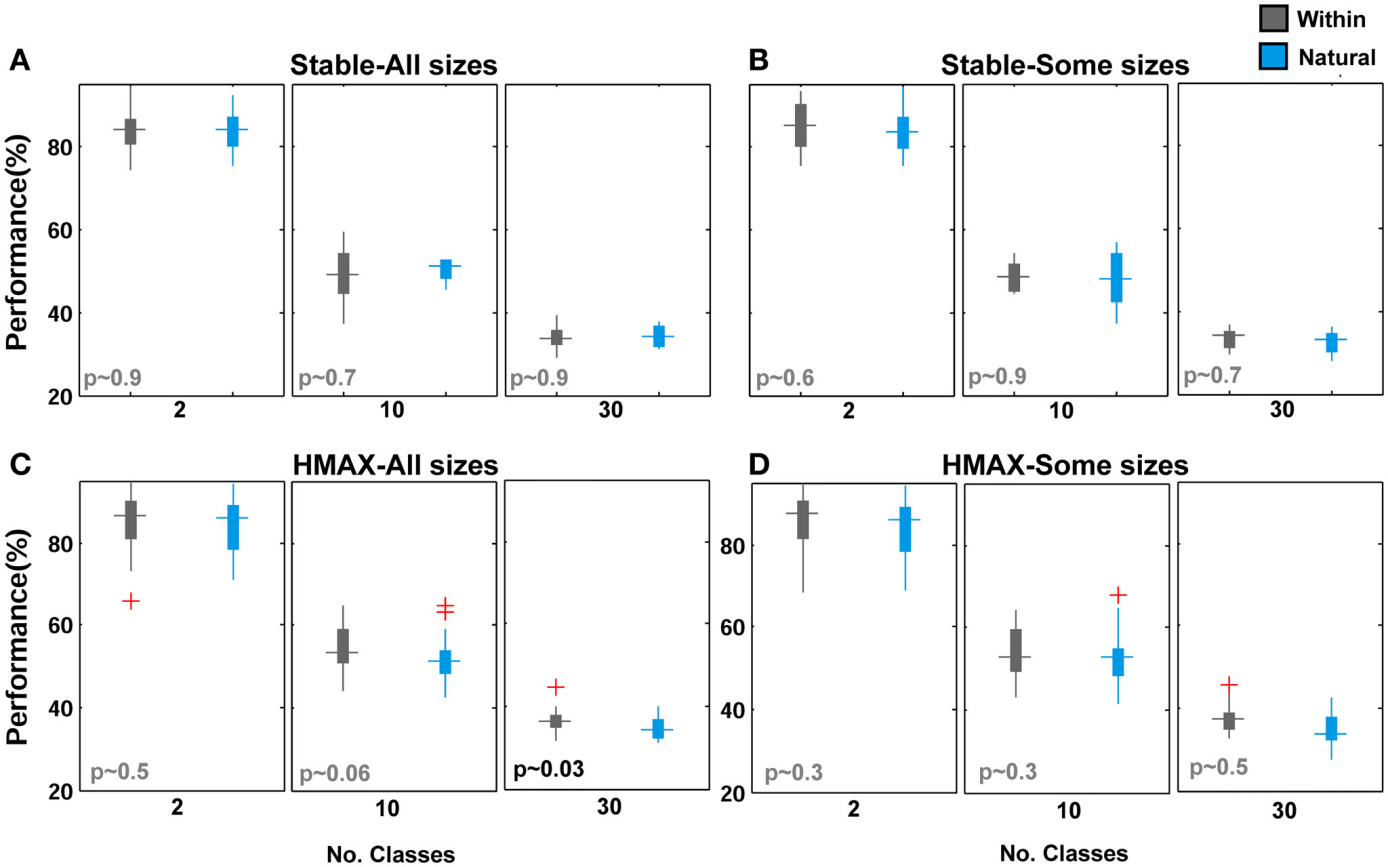
**Performance comparisons between different feature learning strategies in object recognition task. (A)** The performance of the Stable model when all patches contribute to the overall performance in object recognition task for different number of classes. The cyan boxplot shows the performance of the model when feature are learned from natural images and the gray boxplot demonstrates the performance for *within category feature learning* strategy. *P*-values are shown on the left-bottom of each plot. The results are the average of 30 random runs. **(B)** The performance of the Stable model when intermediate patch sizes contribute to overall performance. **(C)** The performance of the HMAX model when all patches contribute to overall performance. **(D)** The performance of the HMAX model when intermediate patch sizes contribute to the overall performance.

To look more closely at performance differences between two modes of feature learning, we averaged the contribution of all patch sizes to the overall performance in two different feature learning strategies. Figures 10A,C demonstrate the performance of the Stable model and the HMAX model respectively, when the contribution of all patches was considered in overall performance. It is obvious that there is no significant difference between class-specific and universal (natural) patches. Further comparisons show that omission of small patch sizes does not make this difference significant, Figures 10B,D. This confirms the idea that using a universal dictionary of visual features is a reasonable approach in generic object recognition. An analogy can also be drawn between this idea and distributed activities of IT neurons in response to different object categories (e.g., Ishai et al., 1999; Haxby et al., 2001; Spiridon and Kanwisher, 2002; O'Toole et al., 2005) in which the patterns of neural activates are distinctive for different object categories. Here, in computational models, the responses of patches to different objects make a pattern of responses in last layer of models that are distinctive to different categories in which a subset of patches from the universal dictionary can be more responsive to a specific object category and other patches show weaker responses to the given category (discussed more in Discussion).

It is also interesting to analyze the representation of different patch sizes in feature space in the generic object recognition. We took a similar approach to face identification task and constructed RDMs for different patch sizes for four object categories but with 50 images from each category for *within feature learning* mode. The results are show in Figure 8 (second row). As illustrated, the contribution of each size is approximately equal in shaping the clusters and feature space.

## Discussion

The mechanisms that the brain uses to perform between and within category object recognition is a fundamental issue in biological object vision. It is not well-understood whether the brain processes faces, which is a well-studied object category, in a different manner from other object or the mechanism is the same for all objects. Since real-world objects have numerous different features and dimensions, this is technically very difficult to explore the representations of these hugely diverse features in the neural response patterns. However, new findings surprisingly indicate that there is significant selectivity in response of some neurons when are visually stimulated with a set of specific objects (e.g., faces) or features of these objects (reviewed in Introduction-e.g., see: Tsao et al., 2003, 2006; Freiwald et al., 2009; Kornblith et al., 2013). Such illustrious discoveries are highly inspiring for computational modelers to theoretically investigate different functions and computations in the neural circuits, particularly visual system and object recognition.

In this study we have argued, taking the advantage of computational modeling, that models can achieve higher performances if they employ a specific set of features and extraction mechanism according to the recognition tasks. Particularly, models required class-specific features with intermediate sizes when performing face identification task (which we here referred to as a within category object recognition), because in this task it is necessary to recognize fine differences between very similar objects. However, this is not the case when models categorized different object categories and a visual dictionary of features yielded to a good performance.

**Figure 11:**
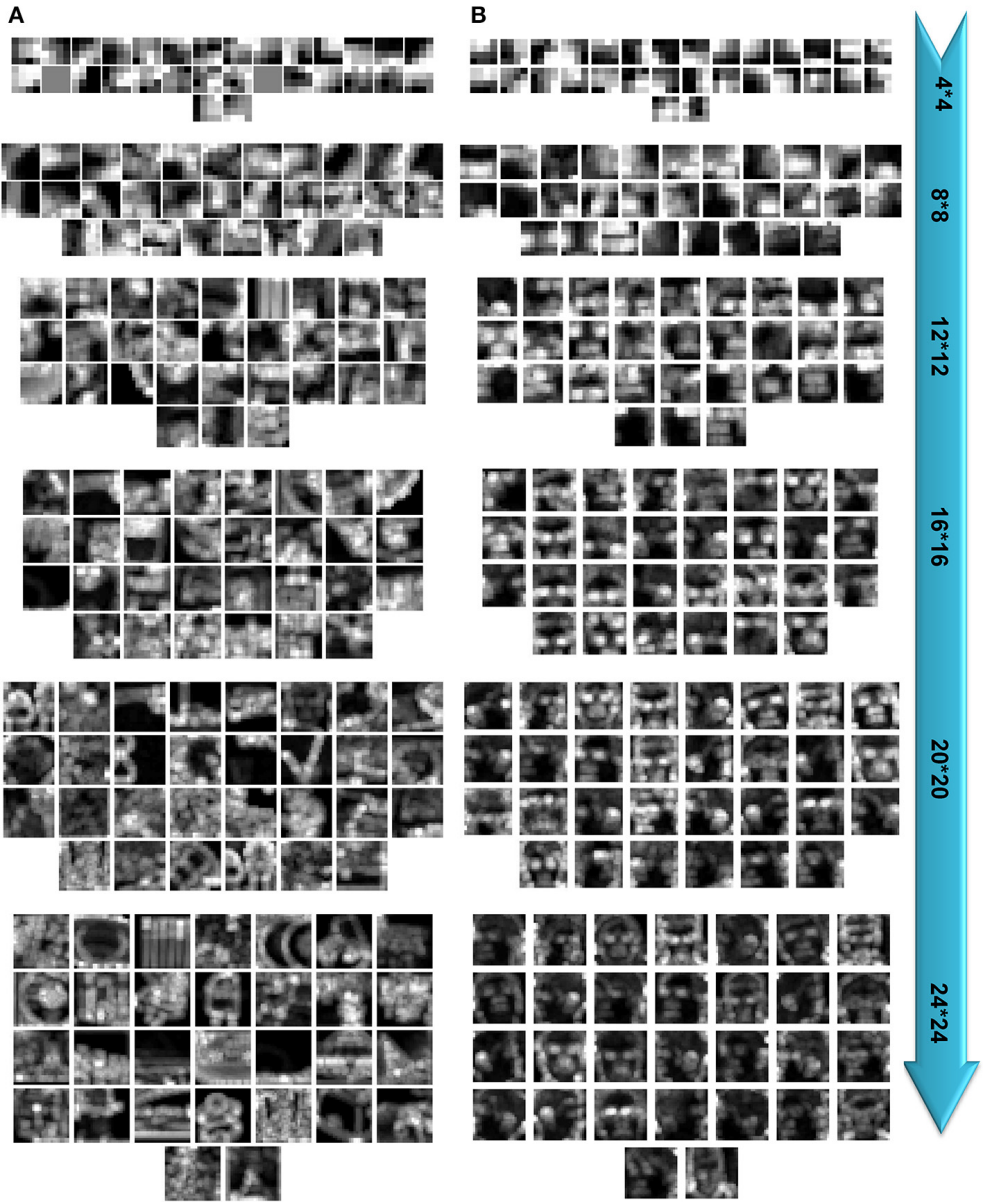
**Samples of extracted patches**. **(A)** Extracted patches in different sizes for object categorization task. **(B)** Extracted patches in different sizes for face identification task.

Our computational simulations showed that face processing needs a different mechanism compared to generic object recognition. The results indicated that the size of visual features (i.e., patch sizes, which has been also called as neural tuning size in some studies-e.g., see Tan and Poggio, 2013) is an important factor for models, probably for visual system, to solve/switch between two recognition tasks (Figure 11 represents samples of extracted patches for face and object images). The performance in face identification task reached to its maximum when models extracted intermediate-size visual features. Increasing the size of features elevates the information content of a feature about a face identity that consequently helps the models to discriminate fine differences between face images more precisely. These results agree to psychophysical studies for face processing that have shown face is holistically processed in the brain. In computational modeling domain, enlarging the size of visual features can helps the models to cover a whole, or partial view of a face with important components (i.e., eyes, nose, month, etc.), and this can be analogous to the concept of holism in visual brain. Although there is ample evidence demonstrating that faces are holistically processed, this is still a very controversial topic in visual recognition, which cannot be fully supported with a model parameter (patch size). However, evidence has shown that such a processing does not happen for other objects. Here we only suggested that face processing seems to be performed more accurately when the size of features is large enough to cover a whole or a partial view of a face. This can be similar to holistic face processing in brain.

We evaluated the performance of two object recognition models: one uses a random mechanism in feature extraction stage (HMAX) and the other employs an unsupervised, biologically inspired mechanism to extract features (Stable). In the HMAX model the number of features for each size must be defined before feature extraction. Therefore, the HMAX model extracted a specific number of features in all experiments (e.g., 250 features with the size of 4^*^4, 250 features with the size of size 8^*^8 and so on). However, the Stable learns a pool of features in an unsupervised manner that the number of learned features can be different from one size to another. This interesting property led us to analyze the number of extracted patches from each size. Interestingly, the Stable model found features with intermediate and large sizes more appropriate in face identification task than other sizes, Figure S3. This result, which was evident in both *within* and *between feature learning* strategies, shows that face identification is solved with intermediate size. In contrast, the number of extracted features with different sizes in object categorization task (except size 4^*^4) is slightly different. These results illustrated the importance of feature size in different recognition tasks.

We have used faces, as a model category, to study within category object recognition. We, however, suggest that for any within category recognition task, models and possibly visual cortex require a different mechanism to separate it from generic object recognition. To differentiate between very similar objects within a specific category, visual brain needs to learn a particular set of features and the feature learning is evolved over a period of time (through development and/or spending a long time experience with a particular object category-e.g., see Johnson and Mervis, 1997; Dahl et al., 2013), depending on the object types. For example, for normal people who see two very similar animals within a biological group (e.g., dogs) for the first time, it might be very difficult to recognize them even after several days or weeks. However, for someone how has spent a long time with the animals (i.e., dog expert), it is a simple task to categorize them (Tanaka and Taylor, 1991) due to a pool of particular features (class-specific) that the owner/expert has learned over time. This is similar to the ability of human adults in face processing (face expert—Tanaka, 2001).

Although our results demonstrated that some properties of visual features (i.e., size, selectivity of features-Figure 11) are important factors in different recognition tasks, we only theoretically investigated the effect of these factors on the performance and object representations using two object recognition models. There are many complex and not very well-understood mechanisms involved in biological object recognition such as the controversies over distributed or localized object representation, semantic or shape-based representation, invariant object recognition, etc. that need to be investigated both computationally and experimentally. This study has simply tried to investigate the role of a model parameter (patch size, which is analogs to neural tuning size) and feature selectivity in two important recognition tasks.

The models used in this study generally had a feed-forward architecture. However, feedback connections between different visual cortical layers and within them change the response dynamic of neurons, and object recognition can be influenced by massive connections coming back from higher areas such as PFC (e.g., Bar et al., 2006; Kveraga et al., 2007). Therefore, inserting feedback connections into the models is interesting for better understanding of object recognition. Moreover, studying object vision and feedback effects in the brain requires looking both at time and space simultaneously (Cichy et al., 2014) since the first flow of visual information is rapidly transformed through feed-forward visual areas (Thorpe et al., 1996; Fabre-Thorpe, 2011) and then feedback projections modulate neural representations in different visual brain areas.

We reviewed some recent studies showing that the FFA (in human, and face patches in monkey) is a part of IT cortex that is specialized for face processing as well as some others results indicate that further to face processing this area are responsible for perceptual expertise (e.g., McGugin et al., 2012) or within category recognition. Modeling the details of this area can uncover some question about mechanism of within category recognition in the brain which can be complementary to experimental studies and provide new experimental ideas.

### Conflict of interest statement

The authors declare that the research was conducted in the absence of any commercial or financial relationships that could be construed as a potential conflict of interest.
