# Supplementary material for: The importance of visual features in generic vs. specialized object recognition: a computational study
Source: Front Comput Neurosci. 2014 Aug 22;8:78. doi: 10.3389/fncom.2014.00078 (PMC4141282; doi:10.3389/fncom.2014.00078)
Supplement: Supplementary file 1 [file Presentation1.ZIP › Supplementary Figures.docx]

**Supplementary Figures:**

**Figure S1. Samples of natural images set that were used in natural feature learning.** The image set includes different environments and scenes. We obtained images by searching on the internet. Sample images, shown here, are randomly selected from natural images in the dataset.

**Figure S2. Samples of object and face images.**

**Figure S3. Number of Selected Patches with the Stable Model in Face identification and Object Recognition.** The number of patches with intermediate and large sizes is significantly higher than small sizes in face identification task. In object categorization selected patches with intermediate sizes are slightly higher than small sizes.
