# Supplementary figures and images for: The importance of visual features in generic vs. specialized object recognition: a computational study
Source: Front Comput Neurosci. 2014 Aug 22;8:78. doi: 10.3389/fncom.2014.00078 (PMC4141282; doi:10.3389/fncom.2014.00078)

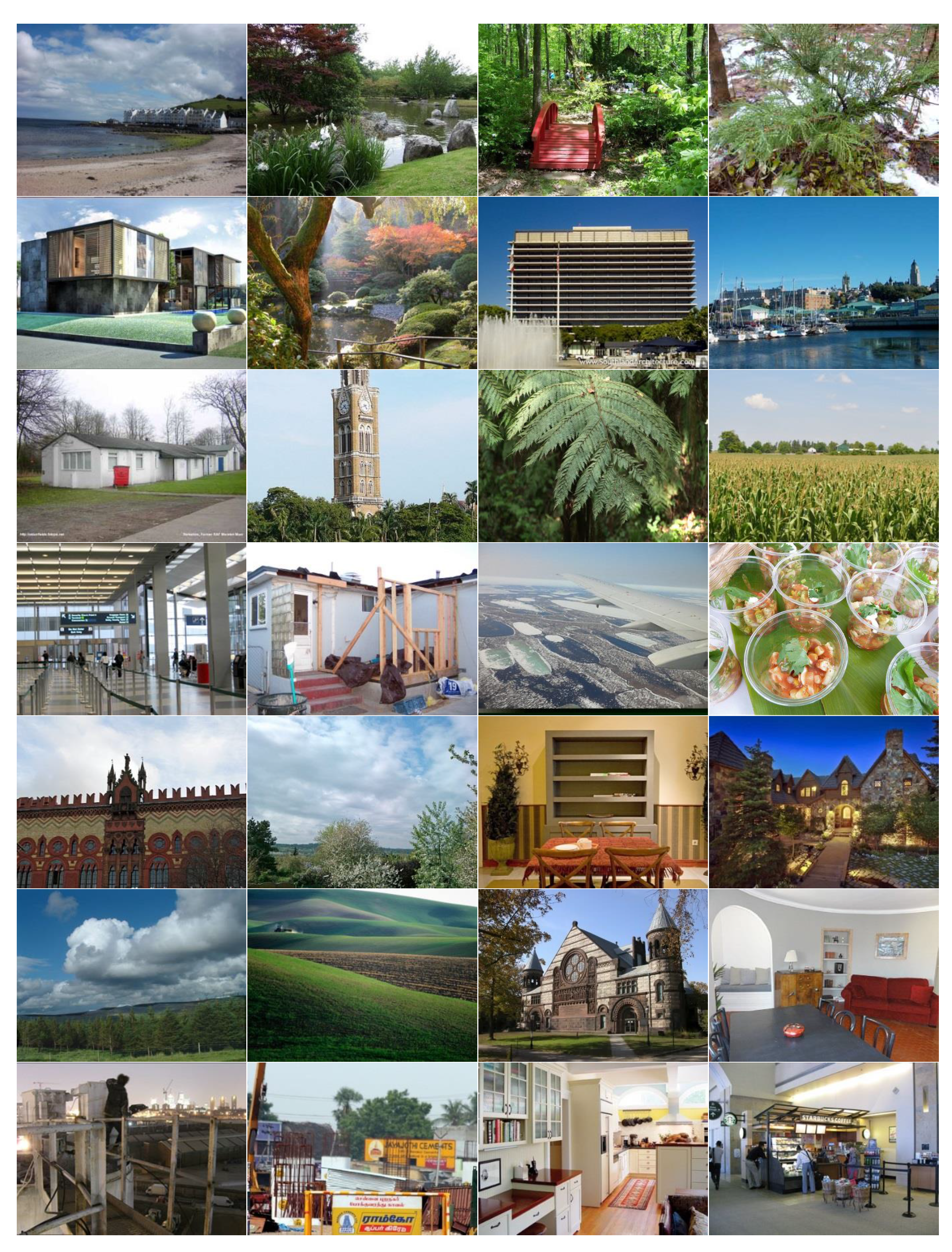

Supplement: Supplementary file 1 [file Presentation1.ZIP › Supp Figure S1.TIF]

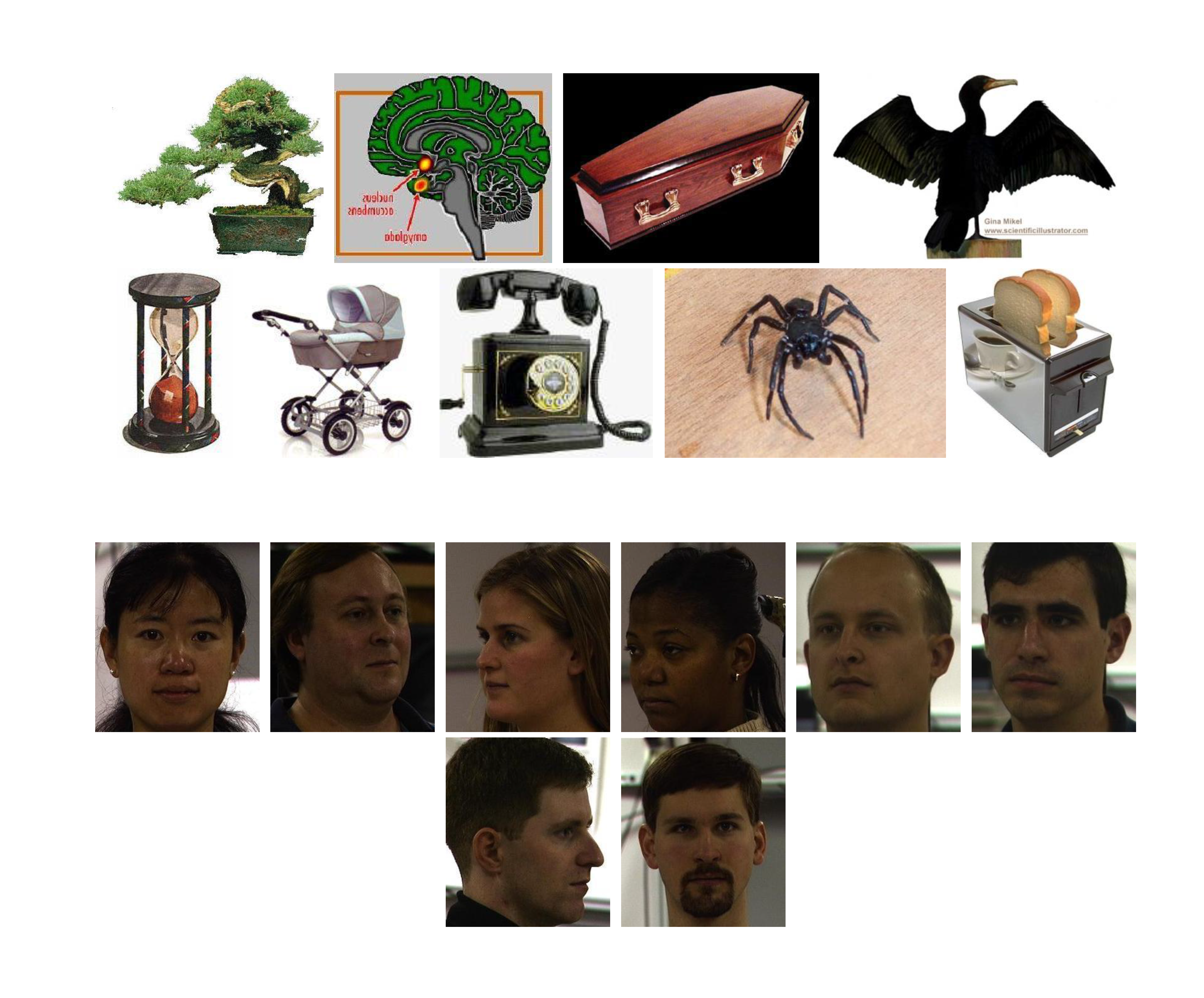

Supplement: Supplementary file 1 [file Presentation1.ZIP › Supp Figure S2.TIF]

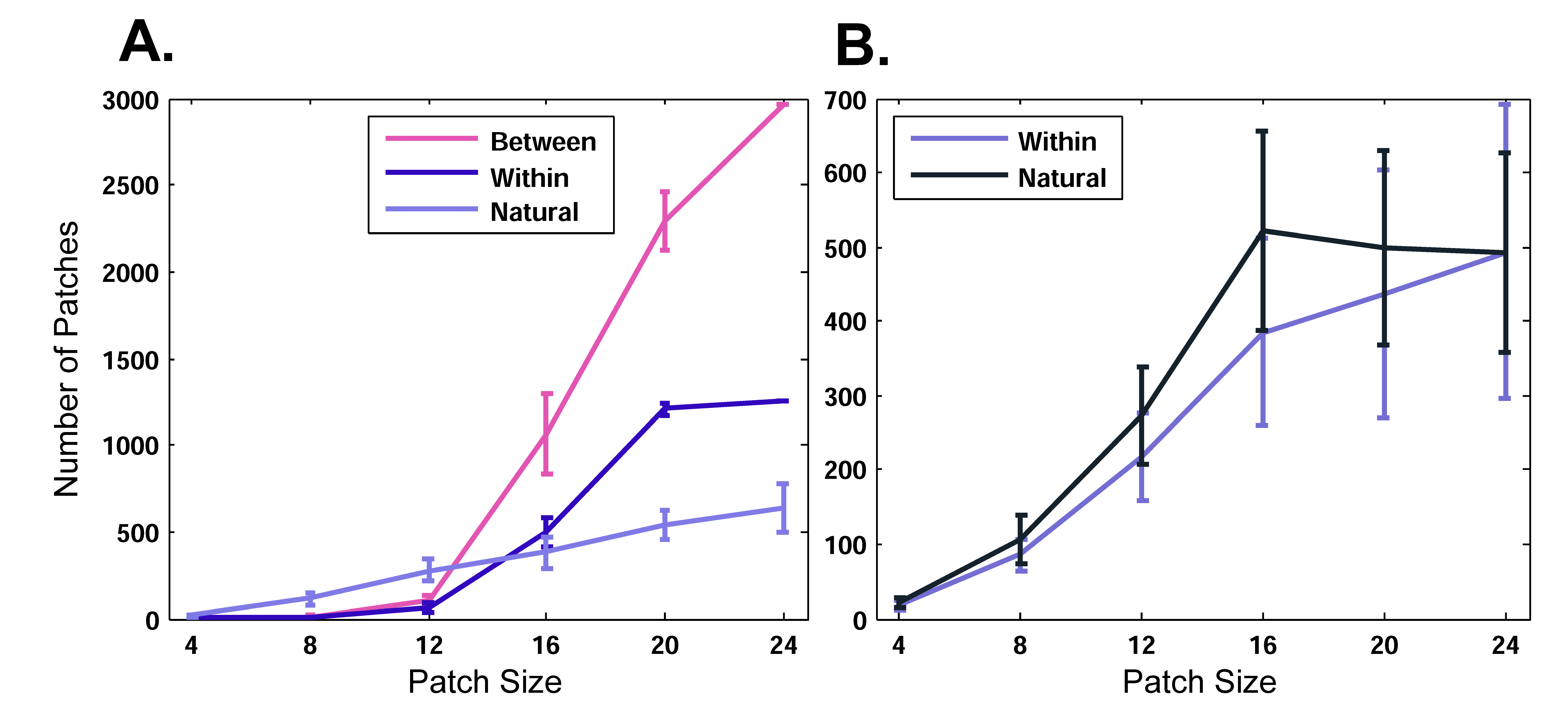

Supplement: Supplementary file 1 [file Presentation1.ZIP › Supp Figure S3.TIF]
